# Brain Gray Matter Volume Associations With Abnormal Gait Imagery in Patients With Mild Cognitive Impairment: Results of a Cross-Sectional Study

**DOI:** 10.3389/fnagi.2019.00364

**Published:** 2020-01-21

**Authors:** Olivier Beauchet, Maxime Montembeault, Gilles Allali

**Affiliations:** ^1^Department of Medicine, Division of Geriatric Medicine, Sir Mortimer B. Davis—Jewish General Hospital and Lady Davis Institute for Medical Research, McGill University, Montreal, QC, Canada; ^2^Dr. Joseph Kaufmann Chair in Geriatric Medicine, Faculty of Medicine, McGill University, Montreal, QC, Canada; ^3^Centre of Excellence on Longevity, McGill Integrated University Health Network, Montreal, QC, Canada; ^4^Lee Kong Chian School of Medicine, Nanyang Technological University, Singapore, Singapore; ^5^Centre de Recherche de l’Institut Universitaire de Gériatrie de Montréal, Montréal, QC, Canada; ^6^Département de Psychologie, Université de Montréal, Montréal, QC, Canada; ^7^Department of Neurology, Geneva University Hospital and University of Geneva, Geneva, Switzerland

**Keywords:** MRI, aged, brain, motricity, EPI-epidemiology

## Abstract

Individuals with mild cognitive impairment (MCI) have worse gait performance compared to cognitive healthy individuals (CHI). The discrepancy between imagined and performed timed up and go test (TUG), known as the TUG delta time, is a marker of brain gait control impairment in individuals with MCI. The study aims to examine the association between the TUG delta time and brain gray matter (GM) volumes in CHI and individuals with MCI. A total of 326 participants, 156 CHI and 170 MCI, with TUG delta time and a brain T1-weighted magnetic resonance imaging (MRI) were selected in this cross-sectional study. Individuals with MCI were older and had greater (i.e., worst performance) performed TUG and TUG delta time compared to CHI. The GM volume association with TUG delta time was examined in CHI and MCI assuming that increased TUG delta time would be associated with locally decreased GM volumes. No significant association was found in CHI, whereas TUG delta time was negatively associated with the GM volume of the right medial temporal lobe in individuals with MCI.

## Introduction

Individuals with mild cognitive impairment (MCI) have worse gait performance compared to cognitive healthy individuals (CHI; Bahureksa et al., 2017; Beauchet et al., 2018). Impairment in gait control at a brain level explains in large part poor gait performance in individuals with MCI (Beauchet et al., 2018). The mental chronometry applied to the timed up and go test (TUG)—the time needed for standing up, walking 3 m, turning, walking back and sitting down—is used to examine impairment in gait control in individuals with MCI (Beauchet et al., 2014). It has been shown that individuals with MCI executed the imagined TUG more quickly than the performed TUG, but not CHI (Beauchet et al., 2014). The discrepancy between imagined and performed TUG, known as TUG delta time, has been proposed as a marker of impairment in gait control at a brain level in individuals with MCI (Beauchet et al., 2010, 2014). No imaging study has examined the association of TUG delta time and brain regions in CHI and individuals with MCI. The hippocampus is a key brain region involved in gait control (Seidler et al., 2010). Decreased hippocampal volume has been reported in individuals with MCI (Tabatabaei-Jafari et al., 2015). Performed TUG has been negatively associated with brain volume reduction in total gray matter (GM) and in the hippocampus in non-demented older adults (Allali et al., 2016). Because both increased TUG delta time and decreased hippocampal volume have been separately reported in individuals with MCI, we hypothesized that increased TUG delta time will be associated with decreased hippocampal volume. The study aims to examine the association between TUG delta time and brain GM volumes in CHI and individuals with MCI.

## Materials and Methods

A total of 326 participants—156 CHI and 170 MCI—referred to the memory clinic of Angers University Hospital (France) were recruited in the “Gait and Alzheimer Interactions Tracking” (GAIT) study. All participants with TUG delta time and a brain T1-weighted magnetic resonance imaging (MRI) were selected in this cross-sectional study. Exclusion criteria were an acute medical illness in the past month, neurological and psychiatric diseases other than cognitive impairment, and medical conditions affecting gait, dementia, and morphological (i.e., dilatation of ventricular system compatible with a diagnosis of normal pressure hydrocephalus) or vascular abnormalities (i.e., stroke) on the brain MRI. Cognitive status (i.e., CHI and MCI) was defined during a multidisciplinary meeting. Information on cognitive performances, physical examination findings, blood tests, and the brain MRI were used. Mini Mental State Examination (MMSE; Folstein et al., 1975), Frontal Assessment Battery (FAB; Dubois et al., 2000), Alzheimer’s Disease Assessment Scale–Cognitive subscale (ADAS-cog; Rosen et al., 1984), Trail Making Test (TMT) parts A and B (Brown et al., 1958), French version of the Free and Cued Selective Reminding Test (Grober et al., 1988; Van der Linden et al., 2004), and Instrumental Activities of Daily Living scale (IADL; Pérès et al., 2006) were the cognitive test used for the assessment of cognitive performance. Participants who had normal neuropsychological and functional performances were considered as cognitively healthy. MCI was defined according to the criteria detailed by Dubois et al. (2010). Participants with any form of MCI, amnestic or non-amnestic and affecting single or multiple domains, were pooled together. Brain imaging was performed with a 1.5 and 3 Tesla MRI scanner (Magnetom Avanto, Siemens Medical Solutions, Erlangen, Germany) following a scanning protocol previously described (Allali et al., 2019). The structural images were processed using voxel-based morphometry (VBM) implemented in SPM12, as previously described (Allali et al., 2019). Overall, the traditional VBM pre-processing steps were conducted, including the creation of study-specific template using the diffeomorphic anatomical registration using exponentiated lie algebra (DARTEL) approach. Angers Ethical Committee (France) approved the study protocol and the recruited participants gave their written informed consent.

The participants’ characteristics were summarized using means and standard deviations or frequencies and percentages, as appropriate. Unpaired *t*-test or Chi-square test was used for the comparisons between CHI and MCI. Whole-brain VBM analyses were conducted to determine the correlations between GM volume association with TUG delta time in CHI and MCI. TUG delta time was entered as a covariate of interest in a multiple regression statistical model including both CHI and MCI individuals entered separately, assuming that increased TUG delta time would be associated with regional decreased GM volumes. Each model was adjusted by age, sex, total intracranial volume, white matter abnormalities and type of MRI. The significance of each effect of interest was determined using the theory of Gaussian fields. Statistical threshold of *P*-value < 0.05 family-wise error (FWE) cluster-corrected was used for all analyses. In addition to correcting for multiple comparisons, a correction for non-stationary smoothness was applied using the implementation of this method in the VBM5 toolbox, which is necessary to avoid false positives or decreased sensitivity when using cluster-size tests (Hayasaka et al., 2004).

## Results

**Figure 1:**
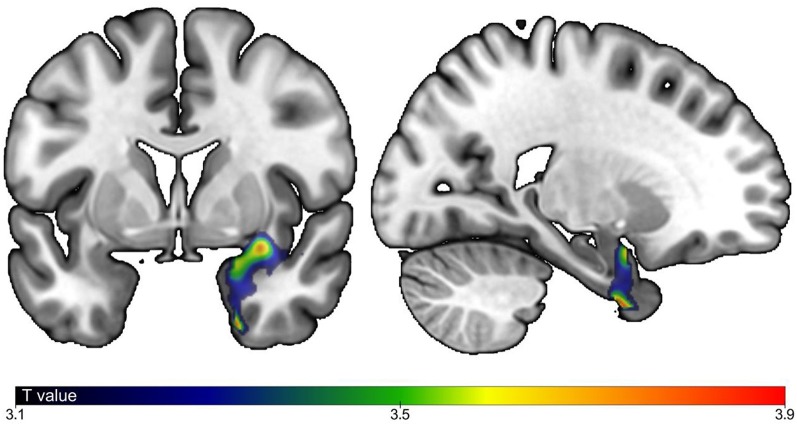
Correlations between brain gray matter (GM) volumes and timed up and go delta time. Correlation between GM regions and gait speed with *P*-Value ≤ 0.05, cluster corrected). Strength of positive association in individuals with mild cognitive impairment (MCI) shown in blue (lowest) to red (highest). Model adjusted by age, sex, total intracranial volume, white matter abnormalities and type of magnetic resonance imaging (MRI).

As shown in Table 1, individuals with MCI were older (*P* = 0.001), had greater performed TUG (*P* = 0.001) and TUG delta time (*P* ≤ 0.001) compared to CHI. There was more male in MCI compared to CHI (*P* = 0.044). There was no significant difference for the other characteristics. The associations of brain GM volumes with TUG delta time are shown in Table 2 and Figure 1. No significant association at the cluster-corrected threshold was found in CHI, whereas TUG delta time was negatively associated with a large medial temporal cluster including the right entorhinal cortex, the amygdala, the parahippocampal gyrus, the insula, and the hippocampus (*P* ≤ 0.05 cluster-corrected) in individuals with MCI. TUG delta time was not associated with GM volume in this region in CHI even when considering the results with an uncorrected threshold.

**Table 1:** Clinical characteristics of participants (*n* = 326).

	Participants		*P*-value*
	CHI (*n* = 156)	MCI (*n* = 170)	
Age (years), mean ± SD	70.4 ± 3.7	70.0 ± 5.1	**0.001**
Male, *n* (%)	81 (51.9)	107 (62.9)	**0.044**
High educational level^‡^, *n* (%)	4 (2.6)	3 (1.8)	0.619
TUG			
Realized (s), mean ± SD	9.5 ± 1.9	10.4 ± 3.1	**0.001**
Imagined (s), mean ± SD	7.3 ± 2.6	6.8 ± 3.0	0.159
Delta time (%)	30.1 ± 27.5	44.4 ± 33.8	**<0.001**
3 Tesla MRI scanner, *n* (%)	60 (38.5)	70 (41.2)	0.617
Total cranial volume (cm^3^), mean ± SD	1,543.1 ± 254.0	1,565.4 ± 323.1	0.494
Total white matter abnormality^||^ volume (cm^3^), mean ± SD	391.4 ± 372.7	454.2 ± 664.3	0.302

MCI, mild cognitive impairment; CHI, cognitively healthy individuals; SD, Standard deviation; *comparisons based on unpaired *t*-test or Chi-square test, as appropriate; ^‡^high school and above; ||defined as MRI signal abnormalities (i.e. T1 hypointensities) and measured using FreeSurfer software; *P*-value significant (i.e. <0.05) indicated in bold.

**Table 2:** Association between gray matter volumes and delta timed up and go time in individuals cognitively healthy and with mild cognitive impairment.

Group of individuals	Cluster/peak regions	Side	*x*	*y*	*z*	Extent	*T*-value*
MCI	Entorhinal cortex	R	21	1	−42	1,024	3.98
	Amygdala	R	31	1	−14	sc	3.77
	Parahippocampal gyrus	R	21	3	−31	sc	3.23
	Insula	R	29	11	−14	sc	3.19
	Hippocampus	R	24	−1	−22	sc	3.15

MCI, mild cognitive impairment; R, Right; L, Left; sc, same cluster; model adjusted by age, sex, total intracranial volume, white matter abnormalities and type of MRI; *P ≤ 0.05 cluster-corrected.

## Discussion

The main finding is that increased TUG delta time was negatively associated with the GM volume of the right medial temporal lobe in individuals with MCI, but not in CHI. This association suggests that TUG delta time may be an appropriate marker of gait control in individuals with MCI; this discrepancy between imagined and performed TUG (i.e., worst gait control) being associated with a decreased GM volume (i.e., worst brain structure) in a key brain region for gait control. This result is consistent with a previous association found between increased gait variability (i.e., worst gait performance) and low hippocampal volume in individuals with MCI and mild dementia (Seidler et al., 2010; Beauchet et al., 2019). Atrophy of the hippocampus is a morphological characteristic of individuals with MCI (Tabatabaei-Jafari et al., 2015). This brain region is a key region involved in memorization and in navigation defined as the ability to move safely in the environment (Tabatabaei-Jafari et al., 2015). TUG delta time may be assimilated as a marker of navigation, low value being an expression of safe navigation and good gait control (Beauchet et al., 2010, 2014). In contrast, increased TUG delta time means inability to navigate appropriately, and this abnormality is associated with abnormality of the brain region controlling navigation. Interestingly, our results were only significant for the right hemisphere. In previous studies focusing on spatial navigation, the right hemisphere and more specifically, the right hippocampus, has been more consistently reported to be related to navigation than the left. For example, in the investigation of navigation skills of London taxi drivers, the correlation between time taxi driving and hippocampal GM volume was right lateralized (Maguire et al., 2006). However, the left hippocampus has been also associated with navigation (Ghaem et al., 1997). The cross-sectional design of this study cannot afford information about the causality of the association between decreased GM volume of the medial temporal lobe and increased TUG delta time, which is the main limitation of our study. Further research needs to examine this association with an observational, prospective, and cohort design with the objective to better understand brain control disorganization in patients with MCI.

## Data Availability Statement

The datasets analyzed in this article are not publicly available. Requests to access the datasets should be directed to olivier.beauchet@mcgill.ca.

## Ethics Statement

The studies involving human participants were reviewed and approved by Angers Hospital ethic committe. The patients/participants provided their written informed consent to participate in this study.

## Author Contributions

All authors listed have made substantial, direct and intellectual contribution to the work, and approved it for publication.

## Conflict of Interest

The authors declare that the research was conducted in the absence of any commercial or financial relationships that could be construed as a potential conflict of interest.
